# Effects of executive functions on consecutive interpreting for Chinese-Japanese unbalanced bilinguals

**DOI:** 10.3389/fpsyg.2023.1236649

**Published:** 2023-08-31

**Authors:** Qichao Song, Ting Song, Xiaodong Fei

**Affiliations:** ^1^Graduate School of Humanities and Social Sciences, Hiroshima University, Hiroshima, Japan; ^2^School of Foreign Studies, Jilin University, Changchun, China; ^3^Beijing Center for Japanese Studies, Beijing Foreign Studies University, Beijing, China

**Keywords:** Chinese-Japanese unbalanced bilinguals, consecutive interpreting, inhibition, updating, shifting

## Abstract

**Introduction:**

Previous research on performance in interpreting has focused primarily on the influence of interpreting experience on executive functions, such as shifting, updating, and inhibition. However, limited research has explored the effects of executive functions on performance. Understanding how different executive functions affect interpreting performance can provide valuable insights for teaching methods. Therefore, the present study aims to examine the effects of executive functions on comprehension and output performance during bidirectional consecutive interpreting between Chinese and Japanese.

**Methods:**

This study involved 48 Chinese advanced Japanese language learners. Self-assessment results indicated that all participants were unbalanced bilingual individuals. All participants took part in consecutive interpreting, completed comprehension tests, and underwent executive function tests. Executive functions were assessed using the color–shape switching task, 1–back task, and Stroop task.

**Results:**

Analysis using Bayesian linear regression revealed the following. (1) Updating exhibited a significant impact on both Japanese-to-Chinese and Chinese-to-Japanese interpreting, indicating that higher updating ability was associated with better interpreting performance. (2) Inhibition showed a significant effect on Japanese-to-Chinese interpreting performance, whereas the effect was not significant in Chinese-to-Japanese interpreting. (3) No significant effects of shifting were observed in either Japanese-to-Chinese or Chinese-to-Japanese interpreting.

**Discussion:**

The results indicate that executive functions have different effects on the interpreting performance of unbalanced bilinguals, while these effects are also influenced by the direction of the source language. Based on these findings, it is recommended that executive function training should be included in interpreter teaching and training programs, with a specific focus on the updating and inhibition functions.

## 1. Introduction

Interpreting is a complex bilingual activity closely linked to the interpreter’s linguistic proficiency and cognitive abilities (Rinne et al., 2000; Henrard and Van Daele, 2017; Song and Li, 2020; Li et al., 2023). Among these cognitive abilities, working memory plays a crucial role in the interpreting process because it has a significant influence on bilingual processing efficiency. Previous research has investigated the relationship between working memory capacity and the quality of interpreting performance (e.g., Tzou et al., 2012; Lin et al., 2018; Bae and Jeong, 2021). However, it has been observed that the later recall in the working memory span test is not an accurate reflection of the actual process of interpreting (Mizuno, 2005). It has been suggested that a more comprehensive exploration of executive functions is necessary to unravel the underlying cognitive mechanisms involved in interpreting (Ma, 2017). Consequently, studying executive functions becomes crucial for gaining a deeper understanding of the cognitive mechanisms underlying the interpreting process.

In recent years, there has been a predominant emphasis on studying the impact of interpreting experience or training on the developmental changes in executive functions (e.g., Yudes et al., 2011; Woumans et al., 2015; van der Linden et al., 2018; Rosiers et al., 2019). In contrast, fewer studies have delved into an understanding of how executive functions specifically influence interpreting performance, particularly in the context of consecutive interpreting (e.g., Dong et al., 2018; Liu and Dong, 2020; Song and Matsumi, 2022). Acquiring a thorough comprehension of the impact of executive functions on interpreting can yield valuable insights into the interplay between individual executive functions and interpreting performance. Such insights would have direct implications for interpreter training, providing valuable guidance for enhancing teaching methodologies. In this light, the present study employs Bayesian linear regression analysis to examine the effects of executive functions on Chinese-Japanese bidirectional consecutive interpreting.

## 2. Literature review

### 2.1. Executive functions in interpreting

“Executive function” refers to a cognitive control mechanism that coordinates diverse cognitive processes to enhance optimal and adaptable performance in complex tasks (Diamond, 2013). Miyake et al. (2000) conducted a study that focused specifically on three commonly postulated executive functions; namely, shifting, updating, and inhibition. They concluded that these functions exhibit moderate correlations with each other while maintaining their distinctiveness. Miyake et al.’s model has served as a framework for exploring the association between executive functions and the comprehension of scientific concepts (Abdullah et al., 2021).

Among the three executive functions, shifting involves the ability to redirect attention flexibly between different tasks or cognitive sets that compete for the same cognitive resources. Updating refers to the ongoing process of actively refreshing and integrating new information into working memory, allowing individuals to adapt to evolving task demands. It involves the ability to replace outdated information with new relevant information. Inhibition, on the other hand, entails the capacity to suppress irrelevant or prepotent responses that may disrupt task performance. It plays a pivotal role in various executive function tasks by inhibiting interference from irrelevant information. For instance, during shifting, inhibition is indispensable in suppressing automatically processed but irrelevant information, whereas during updating it aids in inhibiting outdated and no longer relevant information (St Clair-Thompson and Gathercole, 2006; Zhao and Zhou, 2011; Theodoraki et al., 2020).

As interpreting is a cognitively demanding activity in bilingual contexts, executive functions play a crucial role in ensuring the smooth completion of interpreting tasks (MacNamara et al., 2011; Dong and Li, 2020). Previous research has focused primarily on investigating the impact of interpreting experience or training on executive functions (Nour et al., 2020). The majority of studies have demonstrated that interpreters exhibit superior shifting ability compared to regular bilingual individuals and monolinguals (e.g., Yudes et al., 2011). Interpreter training has been found to improve the shifting ability of student interpreters, as evidenced by reduced switching costs in tasks such as the color–shape switching task (e.g., Dong and Liu, 2016; Zhao and Dong, 2020). Additionally, professional interpreters have shown better updating ability than bilinguals in tasks like the N-back task (Morales et al., 2015), and have demonstrated superior inhibition ability compared to unbalanced bilinguals in tasks such as the Simon task and Attention Network Test (Woumans et al., 2015). Dong et al. (2018) also revealed that interpreter training significantly enhances the development of updating ability in bilingual individuals compared to general second language (L2) training.

Nevertheless, it is important to note that some studies have reported no significant improvement in executive functioning after a period of interpreter training or experience (inhibition: e.g., Morales et al., 2015; shifting: e.g., Babcock and Vallesi, 2017; updating: e.g., Liu and Dong, 2020). These findings suggest that the enhancement of executive functions may be influenced by factors such as the duration and intensity of the training. It is possible that a longer or more intensive training program may be necessary to elicit significant improvements in executive functions related to interpreting.

In comparison to the extensive research exploring the executive function advantages of professional or student interpreters, there have been fewer studies investigating the influence of executive functions on interpreting performance. Moreover, most studies have focused primarily on the impact of updating ability on interpreting performance. Dong et al. (2018) analyzed the impact of executive functions on interpreting performance while also examining the effect of interpreter training on executive functions. Their findings revealed that updating ability significantly predicted interpreting performance. Furthermore, Song and Matsumi (2022) suggested that due to the shared use of ideographic characters (i.e., Chinese characters) between Chinese and Japanese languages, there is a higher probability of non-selective activation of their first language (native language, L1) in bilingual individuals. Therefore, the ability to inhibit non-selective activation would affect interpreting performance. The results of their experiments on Chinese-Japanese interpreting performance using the Stroop task showed that inhibition ability had a significant effect on interpreting performance, particularly in the context of Japanese-to-Chinese interpreting.

In summary, current research on executive functions in interpreting reveals three key points that require further investigation. Firstly, as existing studies have focused primarily on examining the influence of interpreting experience on executive functions, there is a need to explore the impact of executive functions on interpreting performance itself. Understanding how executive functions contribute to interpreting performance would provide valuable insights into the cognitive processes involved. Secondly, whether investigating the impact of interpreting experience on executive functions or examining the influence of executive functions on interpreting performance, the majority of studies have focused predominantly on interpreting between phonographic languages, such as English. There is a notable gap in research on interpreting pairs that involve ideographic characters, such as Chinese and Japanese. Further exploration of executive functions in the context of interpreting between homographic scripts is warranted to gain a more comprehensive understanding of their impact. Thirdly, the majority of studies have focused on individual executive functions, such as shifting, updating, or inhibition, without examining and comparing all three executive functions simultaneously.

### 2.2. Information processing in interpreting

The interpreting process can be divided into three primary stages: source language comprehension, code-switching, and target language output (Gile, 2009; Pöchhacker, 2016). The status of code-switching as an independent stage is a topic of debate, with two opposing perspectives: the serial view and the parallel view. According to the serial view, the retrieval and output of the target language occur only after a complete understanding of the source language. Conversely, the parallel view suggests that lexical units in the target language are accessed and activated prior to a complete comprehension of the source language. These units are subsequently integrated into a coherent discourse representation (Macizo and Bajo, 2004, 2006; Ruiz et al., 2008; Ruiz and Macizo, 2019).

The majority of studies support the parallel view, which suggests that code-switching occurs continuously throughout the process of source language comprehension and target language output, rather than being a distinct stage (Dong and Wang, 2013). However, regardless of when code-switching takes place, the interpreting process can be broadly divided into two main stages: source language comprehension and target language output. Comprehension of the source language serves as the foundation for the target language output (e.g., Lin et al., 2015). It is important to recognize that, unlike general listening comprehension, comprehension of the source language in interpreting, particularly in consecutive interpreting, involves not only a listening stage but also a portion that is integrated into the reformulation and the target language output stages. Comprehension of the source language during the target language reformulation stage is based on the listening stage of the source language.

Regarding the measurement of source language comprehension, sentence interpreting provides a method that involves using the target language output as an indication of the level of source language comprehension (e.g., Fei and Li, 2022; Wang and Fei, 2022). In real-life consecutive interpreting scenarios, especially in consecutive interpreting without note-taking, interpreters rely on contextual cues to understand and memorize the source language before generating the target language output. However, due to limitations in cognitive resources and time constraints in target language output, omissions may occur. This is particularly evident in the alternating interpreting of longer passages, where the likelihood of omissions and even mistranslations is heightened (e.g., Li R. et al., 2022; Song and Matsumi, 2022; Zhang and Cheung, 2022). Consequently, the target language output during consecutive interpreting may not fully reflect a comprehensive understanding of the source language. Following the target language output, comprehension testing can be employed to further explore the depth of source language comprehension.

In contrast to simultaneous interpreting, consecutive interpreting involves a time lag between source language comprehension and target language output. During the source language input stage, interpreters need to allocate their cognitive resources effectively to comprehend the source language and memorize relevant information. During the target language output stage, interpreters must retrieve and reformulate the target language based on the stored source language information. The efficiency of cognitive resource allocation has a significant effect on the performance of comprehension and output in a series of cognitive activities. For example, interpreters with high inhibition ability actively suppress information that may hinder source language comprehension and target language output (Timarová et al., 2014; Song and Matsumi, 2022), while those with high updating ability can quickly remove completed language information from working memory, thus accelerating information processing (Dong et al., 2018; Liu and Dong, 2020). Additionally, interpreters with high shifting ability can complete the transition between the source language and the target language more swiftly, demonstrating better performance (MacNamara et al., 2011; Dong and Li, 2020).

Dong and Lin (2013) pointed out that the interpreting direction has an influence on language processing patterns during interpreting. For unbalanced bilingual interpreters, when the source language is their L1, the overall comprehension of the source language may not be affected by the level of executive functions as the L1 is highly automatized. However, outputting in L2 may impose a significant cognitive load (e.g., Dawrant and Setton, 2016), and the level of executive functions may have a significant impact. Conversely, when the source language is L2, the impact of executive functions may change. This means that the influence of executive functions on source language comprehension and target language output will be closely related to the direction of the source language.

In summary, current research on information processing during interpreting highlights three key points that also require further investigation. Firstly, existing studies of Chinese and Japanese interpreting have focused primarily on sentence-level interpreting. There is a need to explore the information processing mechanisms involved in chapter-level interpreting. Secondly, existing research tends to focus on a single stage of the interpreting process, often emphasizing target language output. There is a lack of comprehensive examination that considers both source language comprehension and target language output as interconnected processes. Thirdly, studies exploring cognitive abilities during the interpreting process have focused predominantly on a single direction, with limited research on the simultaneous consideration of interpreting in different source language directions.

### 2.3. Objectives of this study

Building upon the six key points for exploration mentioned above, the present study aims to investigate the effects of three executive functions on Chinese and Japanese consecutive interpreting based on Miyake et al. (2000)’s “unity and diversity” model. The study specifically targets Chinese-Japanese unbalanced bilinguals and focuses on bidirectional Chinese-Japanese consecutive interpreting within chapter-level contexts. The study aims to address two primary research questions:

**RQ1:** How do updating, inhibition, and shifting affect scores of source language comprehension and target language output during Japanese-to-Chinese consecutive interpreting?

**RQ2:** How do updating, inhibition, and shifting affect scores of source language comprehension and target language output during Chinese-to-Japanese consecutive interpreting?

By addressing these research questions, this study aims to enhance our comprehension of how executive functions influence interpreting performance, thus providing valuable theoretical insights for interpreter training.

## 3. Materials and methods

### 3.1. Participants

The participants totaled 48 Japanese language learners from China, including 36 females. They had an average age of 22.69 years (*SD* = 1.76) and had studied Japanese for an average of 5.94 years (*SD*  = 1.75). At the time of the experiment, all participants had attained the highest level in the Japanese Language Proficiency Test (JLPT N1), with an average score of 146.58 (*SD*  = 22.03) out of a total possible score of 180. All participants have been studying Japanese since their time as undergraduates or high school students. No participant had experienced living in Japan for an extended period before reaching adulthood. Table 1 presents the participants’ self-assessment scores for their bilingual ability and usage frequency (as proposed by Li et al., 2020). The self-assessment results indicated that all participants were unbalanced bilinguals. None of the participants had received professional interpreter training. Upon completion of the experiment, participants received a specified compensation.

**Table 1:** Self-assessment and comparison of participants’ bilingual ability and usage frequency in Chinese and Japanese.

		Chinese (L1)	Japanese (L2)	Comparison (*t-*test)
Bilingual ability	Listening	8.92 (0.96)	7.00 (0.95)	*t* (47) = 14.46^***^
	Speaking	8.04 (1.25)	6.44 (1.17)	*t* (47) = 9.28^***^
	Reading	9.10 (0.83)	7.88 (1.30)	*t* (47) = 7.77^***^
	Writing	8.00 (1.07)	6.44 (1.20)	*t* (47) = 8.76^***^
Usage frequency	Time of usage	11.14(4.22)	5.58(2.06)	*t* (47) = 9.46^***^
	Thinking	6.31(0.75)	3.31(1.21)	*t* (47) = 14.39^***^
	Talking to yourself	5.71(1.09)	3.29(1.25)	*t* (47) = 8.97^***^
	Expressing emotion	5.92(1.18)	3.69(1.67)	*t* (47) = 6.64^***^
	Dreaming	6.46(0.92)	1.85(1.30)	*t* (47) = 18.79^***^
	Arithmetic	6.67(0.69)	1.50(0.90)	*t* (47) = 24.96^***^
	Remembering numbers	6.75(0.60)	1.19(0.45)	*t* (47) = 45.44^***^

^***^p < 0.001. Results are expressed as mean (SD). Bilingual ability: 1, very poor ~ 10, excellent. Time of Usage: hours/per day. Usage frequency: 1, never ~ 7, always.

### 3.2. Design

Liu et al. (2021) have highlighted the issue of small sample sizes in current studies on interpreting, which hampers the ability to draw definitive conclusions. In this study, given the emphasis on cognitive factors, controlling for variables such as participants’ language learning background and proficiency made it relatively challenging to obtain a large sample size. Using structural equation modeling (SEM) analysis under these circumstances may lead to decreased model fit and reduced reliability of the study (Kline, 2005). To address this limitation, we utilized commonly used methods for assessing executive functions from previous studies (e.g., Timarová et al., 2014; Dong and Liu, 2016) and treated them as observed variables in our analysis. Additionally, Bayesian statistics offer a solution to the issue of small sample sizes by effectively utilizing sample information through random sampling and obtaining robust parameter estimates, even with limited samples (e.g., McNeish, 2016; Zhong et al., 2017).

Therefore, in this study, we employed Bayesian linear regression analysis to examine the influence of executive functions on Chinese-Japanese bidirectional consecutive interpreting. The study considered the scores of source language comprehension and target language output as dependent variables, while shifting, updating, and inhibition were treated as independent variables. By utilizing Bayesian statistics, our study aims to provide more reliable and informative insights into the relationship between executive functions and Chinese-Japanese bidirectional consecutive interpreting.

### 3.3. Materials

#### 3.3.1. Source language and comprehension test items

To ensure consistent levels of difficulty, a double-verification process was implemented for the materials used in this experiment. Since all participants were advanced learners without professional interpreter training, source language materials relevant to their daily life and learning were selected from Lin (2019). The Chinese Readability Index Explorer (CRIE 3.0)^1^ was used to confirm the text difficulty of the Chinese-to-Japanese materials and the Japanese-to-Chinese reference translations. Additionally, the Japanese-to-Chinese materials and the Chinese-to-Japanese reference translations were assessed for difficulty using the jReadability Portal.^2^ This double-verification process ensured that the materials used in the experiment had consistent levels of difficulty.

To further validate the selection of materials, a group of 28 advanced Japanese learners, who had a similar level of Japanese proficiency as the participants in this study, were asked to assess the difficulty and familiarity of the materials. The assessment was conducted using a 7-point rating scale. A *t*-test was performed on the rating values, and the results indicated no significant difference in difficulty and familiarity between the two sets of materials [*t*(27) = 1.44, *p* = 0.161, *d* = 0.27]. Furthermore, the 48 participants evaluated the familiarity and difficulty of the materials after the experiment had concluded. The results showed no significant differences between the two sets of materials [*t*(47) = 0.73, *p* = 0.472, *d* = 0.10]. The evaluation results and readability indices can be found in Table 2. The Japanese materials were recorded by a Japanese native speaker specializing in Japanese language education, while the Chinese materials were recorded by a Chinese native speaker with broadcasting experience. To ensure semantic coherence, the recordings were segmented into meaningful units using audio-visual processing software. On average, each segment contained approximately 5 meaningful units (similar to a simple sentence, see Supplementary material). The segmentation process took into consideration Lin (2019)’s principles of segmentation and the use of conjunctions in the article.

**Table 2:** Summary of characteristics of the source language.

		J-C	C-J
Duration		5′01”	4′38”
Segment count		6	5
Meaningful unit count/per segment		5.17 (1.17)	5.00 (0.71)
Difficulty rating	Pretest (*N* = 28)	2.18 (1.19)	1.96 (0.88)
	Posttest (*N* = 48)	3.45 (0.90)	3.56 (0.94)
Summary of Chinese text analysis results	Character count	323	319
	Word count	175	173
	Sentence count	19	18
	Average word count/per sentence	9.21	9.61
	Lexical Density	0.79	0.79
Summary of Japanese text analysis results	Character count	547	528
	Word count	336	309
	Sentence count	15	13
	Average word count/per sentence	22.40	23.77
	Difficulty	Intermediate level	Intermediate level

Results are expressed as mean (*SD*). J-C, Japanese-to-Chinese consecutive interpreting. C-J, Chinese-to-Japanese consecutive interpreting. The summary of the Chinese text analysis results is based on CRIE 3.0. For J-C materials, its Chinese reference translations were used. The summary of the Japanese text analysis results is based on jReadability. For C-J materials, its Japanese reference translations were used. The criteria for determining readability level are based on the assessment of difficulty level for Japanese learners, taking into account sentence length and lexical density.

In addition, to assess participants’ comprehension, a cued recall task was administered after the interpreting test to measure their understanding of the source language. The decision to use a cued recall task was driven primarily by the objective of minimizing forgetting during free recall and maximizing participants’ ability to recall the comprehended content. The cues for the questionnaire items were developed through collaboration between a native Japanese teacher and a native Chinese teacher. They identified the essential content in the two sets of materials, which served as the foundation for designing the comprehension test items. To further refine the comprehension questions, a pretest was conducted with three advanced learners and their feedback was taken into account. The evaluation criteria for the comprehension test were established based on the essential content selected by the two teachers. For Japanese-to-Chinese interpreting, there were 11 key content items, while for Chinese-to-Japanese interpreting there were 8 key content items. An example of the experimental materials can be found in Table 3.

**Table 3:** Examples of experimental materials.

	Source language	Comprehension test item
J-C	では, これから講座をお聞きいただくにあたって, いかに学習をしていくとよいか, 気をつけていただきたいことなどをお話ししましょう。次の三点をお願いしたいと考えております。(Now, let me talk about how you can approach your learning and highlight some important points to consider before attending the lecture. I would like to emphasize the following three points.)	What is the main content of the lecture?
C-J	现在, 年轻人都玩手机, 还用它买衣服, 吃的, 用的啊等等, 干什么都喜欢使用手机。只要自己选好想要的东西, 轻轻一点, 交易就算完成了, 你说这手机方便不方便?(Nowadays, young people are all using their mobile phones for everything, from buying clothes to ordering food and making various other transactions. They prefer using their mobile phones for everything. With just a gentle touch after selecting the desired item, the transaction is completed. Can you imagine how convenient these phones are?)	What do young people do with mobile phones these days? How do mobile phones demonstrate their convenience?

J-C, Japanese-to-Chinese consecutive interpreting. C-J, Chinese-to-Japanese consecutive interpreting.

#### 3.3.2. Assessment of executive functions

The executive function tests were programmed using the open-source package PsychoPy (version 2021.2.3, Peirce, 2007). Shifting was assessed using the color–shape switching task (e.g., Babcock and Vallesi, 2017). Updating was evaluated using the digital 1–back task (e.g., Harvey et al., 2004), and inhibition was tested using the classical color–word Stroop task (e.g., Stroop, 1935). Before beginning each task, participants underwent practice exercises specifically designed for each task to ensure that they were familiar with the experimental procedure. Response keys were labeled to facilitate quick responses during the testing phase.

##### 3.3.2.1. Shifting

The color–shape switching task was divided into three blocks: a color task block with 30 trials, a shape task block with 30 trials, and a mixed task block with 40 trials. The stimuli possessed both color (blue or red) and shape (triangle or circle) attributes. In the color task, participants were required to ignore the shape and judge the color (blue: “←” key; red: “→” key). In the shape task, participants were told to ignore the color and judge the shape (triangle: “←” key; circle: “→” key). In the mixed task, participants were required to follow instructions on the computer screen (“please sort by shape”; “please sort by color”) before making each judgment. In mixed tasks, the difference in average response times between consistent conditions (20 trials, where both the former and latter judgment rules are shapes or colors) and inconsistent conditions (20 trials, where the former judgment rule is “shape” and the latter is “color,” or vice versa) served as an indicator of participants’ shifting abilities. A smaller difference indicates higher shifting ability.

##### 3.3.2.2. Updating

The digital 1–back task consisted of a total of 80 trials. Random numbers were displayed at the center of the computer screen, and participants were required to determine whether the current number matched the previously presented number. Participants were instructed to press the “L” key when the numbers matched and the “K” key when they did not. The measurement index for this task was the average response time for accurately identifying the match, with shorter response times indicating a higher updating ability.

##### 3.3.2.3. Inhibition

The stimuli used in the color–word Stroop task included the Chinese characters “红” (red), “黄” (yellow), “蓝” (blue), and “绿” (green). The task consisted of a total of 80 trials, with 40 trials in the color–word consistent condition and 40 trials in the inconsistent condition. In the consistent condition, the color of the Chinese character matched its semantic meaning (e.g., the word “黄” written in yellow font color). In the color–word inconsistent condition, the color of the Chinese character was inconsistent with its semantic meaning (e.g., the word “红” written in yellow font color). Participants were instructed to ignore the meaning of the Chinese characters and focus on judging their color. The keys “J,” “K,” “F,” and “D” on the computer keyboard corresponded to the colors “red,” “yellow,” “blue,” and “green,” respectively. The measurement index for this task was the difference between the average response time for accurately judging colors in inconsistent and consistent conditions. A smaller difference indicated higher inhibition ability.

### 3.4. Procedure

**Figure 1:**
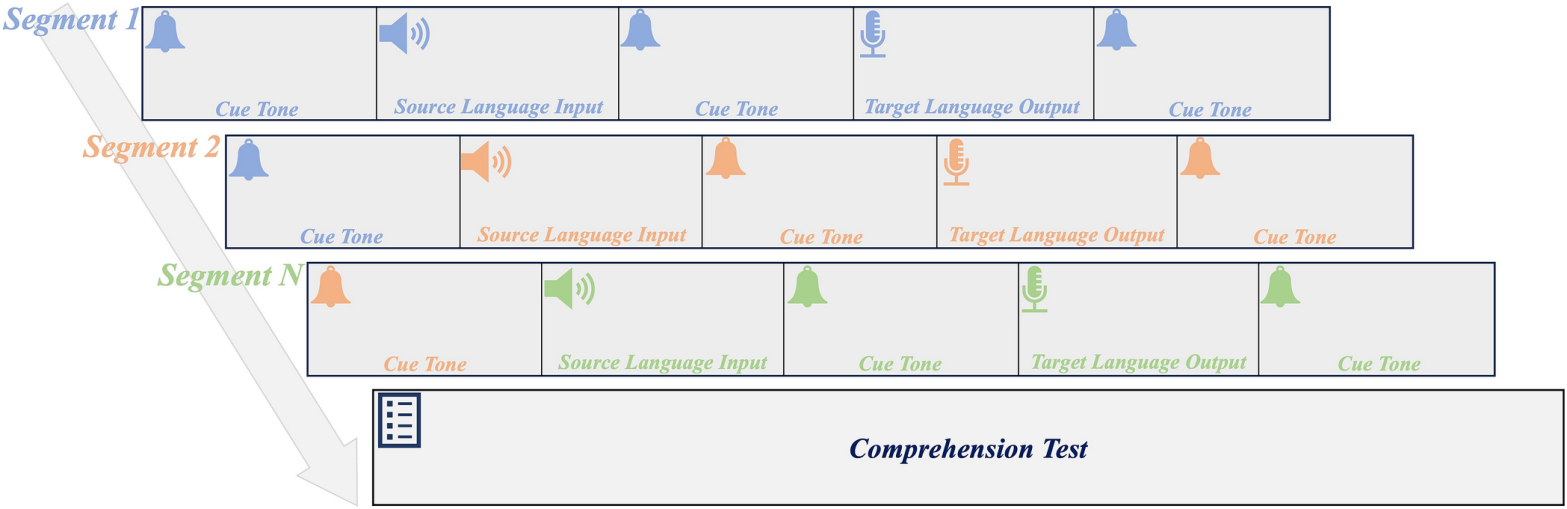
The procedure of one consecutive interpreting test.

The experiment consisted of three main components: consecutive interpreting and comprehension tests, executive function tests, and post-experiment questionnaires. The consecutive interpreting tests were based on the format of the China Accreditation Test for Translators and Interpreters (CATTI). The process of consecutive interpreting is illustrated in Figure 1. Initially, participants listened to a cue tone through headphones, followed by the auditory presentation of a segment in the source language. Subsequently, another cue tone signaled the start of the interpreting process. Participants were then required to generate the corresponding target language until another cue tone was played. The participant’s output was recorded for further analysis.

Following the completion of one consecutive interpreting session (either from Chinese to Japanese or from Japanese to Chinese), participants were instructed to complete a comprehension test accordingly. In this test, participants were required to provide to the best of their ability detailed answers to each question in Chinese. The order of the two consecutive interpreting tests was counterbalanced to minimize any potential order effects.

After completing the two consecutive interpreting tests, participants proceeded to the executive function tests. The order of the three executive function tests was counterbalanced to avoid any potential order effects that might influence a participant’s performance. Subsequently, participants were given questionnaires to collect information about their background in Japanese language learning and prior exposure to or practice with interpreting test materials. Additionally, a questionnaire was administered to assess the participant’s perception of the level of difficulty of the experimental materials.

## 4. Results

### 4.1. Data processing

#### 4.1.1. Source language comprehension score

To score their source language comprehension, participants’ written answers were evaluated using predetermined criteria. Participants’ answers were checked against the predetermined answers to each question, and a point was awarded if their answer matched the predetermined answer. The scores for each material were calculated by dividing the correct number of participants’ written answers by the total number of key contents. These scores represented the comprehension scores for the respective materials and were utilized in the final analysis.

#### 4.1.2. Target language output score

Following the evaluation criteria established by Yang (2005), two Chinese Japanese language teachers with expertise in Japanese language education assessed source language output on a 100-point scale. We utilized Yang (2005)’s evaluation sheet as it is widely used for evaluating interpreting performance and has undergone multiple reliability and validity checks (e.g., Han and Fei, 2021; Li J. et al., 2022; Zhang and Liu, 2023). The participants’ outputs were evaluated based on a scoring system that allocated 50 points for fidelity (presence of mistranslations and omissions), 30 points for delivery (clarity and fluency), and 20 points for language use (grammar and vocabulary selection).

Before the scoring process, the two evaluators were provided with explanations of the three micro-level evaluation items, following Yang (2005)’s guidelines. Each evaluation item was assigned a scoring range, consisting of “poor (completely below criteria),” “limited (only partially meets the criteria),” “average (meets more than half of the criteria),” “good (basically meets the criteria),” and “excellent (almost fully meets the criteria).” For example, in the case of fidelity, the scoring intervals were defined as follows: “poor (0–10), limited (11–20), average (21–30), good (31–40), and excellent (41–50).” The evaluators initially determined the level for each item and then provided scores within that range.

After the scoring was completed, we performed a reliability analysis and a correlation analysis. An explanation of Yang (2005)’s evaluation criteria and the results of the correlation and reliability analyses are presented in Table 4. The reliability analysis revealed high Cronbach’s alpha coefficients, indicating that the evaluation criteria were effective. Furthermore, the correlation analysis showed a strong positive correlation for both source languages, indicating a high level of agreement between the two evaluators’ ratings. The final scores for the participants’ interpreting output were determined by calculating the average of the scores provided by the two evaluators.

**Table 4:** Explanation of evaluation criteria, summary of correlation analysis and reliability analysis results.

Check point	Mean score and Pearson’s correlation						Cronbach’s alpha
	J-C			C-J			
	E1	E2	*r*	E1	E2	*r*	
***Fidelity*** 1) Are there any mistakes in understanding the content (including specific nouns)? 2) Are there any omissions in the translation? 3) Is the translation an over-translation (i.e., more than the original content)?	37.10 (4.70)	35.23 (4.05)	0.86^***^	36.27 (4.98)	35.15 (4.17)	0.75^***^	0.864
***Delivery*** 1) Clarity: a) Are the divisions and connections between sentences and paragraphs clear? b) Is the pronunciation (accent, intonation) clear? 2) Fluency: a) Are there any hesitations or pauses longer than 2 s? b) Are there many repetitions of the same phrases?	22.25 (2.11)	21.67 (2.34)	0.74^***^	21.50 (2.25)	21.40 (1.61)	0.73^***^	0.766
***Language use*** J-C: Is the selection of expressions appropriate? C-J: Are the vocabulary choices and grammar usage	12.79 (1.82)	12.23 (1.89)	0.76^***^	11.83 (1.64)	11.81 (1.39)	0.72^***^	0.777

^***^p < 0.001. J-C, Japanese-to-Chinese consecutive interpreting. C-J, Chinese-to-Japanese consecutive interpreting. E1, Evaluator 1. E2, Evaluator 2. Results are expressed as mean (*SD*).

#### 4.1.3. Data curation of executive function tests

Incorrect response times were excluded from the analysis for all three tasks: shifting task (exclusion rate: 5.12%); updating task (exclusion rate: 3.05%); and inhibition task (exclusion rate: 3.54%). Additionally, data points that deviated from the mean by more than ±2.5*SD*s were also excluded: shifting task (exclusion rate: 3.85%); updating task (exclusion rate: 1.90%); and inhibition task (exclusion rate: 2.68%).

As mentioned earlier (Section 3.2.2), the evaluation index for shifting ability was determined from the color–shape task and by calculating the difference in response times between the inconsistent conditions (Mean = 888.60 ms; *SD* = 194.49) and the consistent conditions (Mean = 770.38 ms; *SD*  = 191.36). The evaluation index for updating ability was obtained as the mean response time in the digital 1–back task (Mean = 567.64 ms; *SD*  = 66.97). The evaluation index for inhibition ability was determined from the color–word task and by calculating the difference in response times between the inconsistent conditions (Mean = 1128.14 ms; *SD* = 182.28) and the consistent conditions (Mean = 972.96 ms; *SD*  = 152.13). Table 5 presents descriptive statistics and the correlation matrix displaying the scores for source language comprehension, target language output, and the results of each executive function test.

**Table 5:** Descriptive statistics and correlation matrix between the interpreting tasks and the indicators of the three executive functions.

	Mean	*SD*	1	2	3	4	5	6	7
1. J-C comprehension score	62.12	14.57	1.00						
2. C-J comprehension score	79.43	12.23	0.22	1.00					
3. J-C output score	70.64	7.81	0.50^***^	0.19	1.00				
4. C-J output score	68.98	7.06	0.22	0.37^**^	0.55^***^	1.00			
5. Shifting	118.22	100.07	−0.24^†^	−0.17	−0.53^***^	−0.42^**^	1.00		
6. Updating	567.64	66.97	−0.06	−0.19	−0.45^**^	−0.54^***^	0.34^*^	1.00	
7. Inhibition	155.18	158.15	−0.34^*^	−0.26^†^	−0.67^***^	−0.43^**^	0.47^***^	0.24^†^	1.00

^†^*p* < 0.10, ^*^p < 0.05, ^**^p < 0.01, ^***^p < 0.001. J-C, Japanese-to-Chinese consecutive interpreting. C-J, Chinese-to-Japanese consecutive interpreting.

### 4.2. Correlation between fidelity score and source language comprehension score

In the light of previous research which suggests that target language output alone may not be an accurate reflection of the level of source language comprehension in consecutive interpreting (Song, 2022), this study included tests to assess overall comprehension levels. Specifically, we employed source language comprehension tests conducted after the target language output, which, from a temporal perspective, encompassed both the source language input and the target language output stages. Thus, it was essential to explore whether the comprehension scores could effectively reflect participants’ comprehension during the source language input stage. In this section, we began by examining the relationship between scores on the source language comprehension test and fidelity scores (averaged scores from two evaluators) in the evaluation of target language output. This analysis allowed us to verify the existence of instances where participants accurately comprehended the source language but encountered challenges in effectively reproducing it in their target language output.

We hypothesized that if participants continued to comprehend the source language during the reformulation and target language output stages, the memory trace of the content would deepen. Given that our study employed consecutive interpreting without note-taking, it was highly probable that content with stronger memory traces would be more accurately produced. By assessing the strength of the correlation between fidelity scores and comprehension scores and examining the results of the regression analysis, we could determine to what extent the comprehended content had been accurately produced. If a significant correlation and a strong predictive relationship were found between these measures, we could conclude that the comprehended content had indeed been faithfully outputted. Conversely, if there was no strong correlation between fidelity scores and comprehension scores, it would suggest that although the offline comprehension scores represented a combined score of both comprehension and output stages, the likelihood of further comprehension during the reformulation and output stages was relatively low. In other words, we can infer that these scores primarily reflected participants’ understanding of the source language input stage rather than their reformulation and target language output stages.

The results of the correlation analysis indicate a significant moderate positive correlation in both Japanese-to-Chinese interpreting (*r* = 0.52, *p* < 0.001) and Chinese-to-Japanese interpreting (*r* = 0.36, *p* = 0.012), with a lower correlation coefficient observed in the latter case. Additionally, separate simple linear regression analyses were conducted using fidelity scores as the independent variable and comprehension scores as the dependent variable. The findings demonstrate that fidelity scores significantly predict comprehension scores in both Japanese-to-Chinese interpreting (adj. *R^2^* = 0.25) and Chinese-to-Japanese interpreting (adj. *R^2^* = 0.11), although the explanatory power of the models is relatively modest. These findings suggest that in chapter-level interpreting, using the interpreting output to infer the comprehension of the source language may result in certain biases. In contrast, comprehension scores may provide a more accurate reflection of participants’ understanding, and it can be argued that these scores primarily indicate the level of comprehension during the source language input stage.

### 4.3. Results for the effect of executive functions on consecutive interpreting

#### 4.3.1. Analysis methods

In this study, Bayesian linear regression analysis was conducted on the collected data using R (version 4.2.1, R Core Team, 2022) and the *brms* package developed by Bürkner (2017). Priors over parameters were set as student *t*-distributions. The Markov Chain Monte Carlo (MCMC) method was employed for sampling simulations.

The MCMC sampling process involved running 4 chains. Each chain consisted of a total of 2000 sampling iterations, including a warmup period of 1,000 iterations. A thinning interval of 1 was used. The convergence of the sampling process was assessed using the Rhat statistic, which measures the potential scale reduction factor. A value of 1.00 for Rhat indicates convergence of the 4 chains, suggesting that the sampling process has stabilized. The regression model used in this study can be summarized as follows:


$$y_{i}=\beta_{0i}+\beta_{1i}*\text{shifting}+\beta_{2i}*\text{updating}+\beta_{3i}*\text{inhibition}+\mu_{i}$$

The dependent variable, represented as $y_{i}$, refers to the comprehension or output scores of participant *i*. The regression model includes an intercept $\beta_{0}$ that represents the baseline. The coefficients $\beta_{1}$, $\beta_{2}$, and $\beta_{3}$ describe the effects of the shifting, updating, and inhibition functions, respectively. The term *μ* represents the error term.

Following the methodology of previous studies, if the 95% Bayesian credible interval of a parameter excludes the value 0, it indicates a significant effect of the factor. The methodology is similar to the conventional hypothesis testing employed in frequentist statistics (e.g., Namba et al., 2018).

#### 4.3.2. Analysis results of Japanese-to-Chinese consecutive interpreting

The results of the Bayesian linear regression analysis indicated that executive functions had different levels of explanatory power for the source language comprehension scores and target language output scores in Japanese-to-Chinese interpreting. For the comprehension scores, the executive functions showed a weak explanatory power (*R^2^* = 0.15, 95% CI [0.02, 0.30], adj. *R^2^* = 0.02). However, for the output scores, the executive functions demonstrated a substantial explanatory power (*R^2^* = 0.56, 95% CI [0.42, 0.67], adj. *R^2^* = 0.49).

Table 6 provides a summary of the analysis results. Regarding the source language comprehension scores, none of the three executive functions showed a significant effect. Specifically, shifting (*β* = −17.52 [−64.04, 30.73]), updating (*β* = 11.85 [−54.34, 76.49]), and inhibition (*β* = −27.17 [−55.98, 1.29]) did not reach statistical significance concerning the comprehension scores. However, when examining the target language output scores, the results revealed a significant effect of updating (*β* = −29.97 [−55.62, −4.53]). Higher updating ability was associated with higher output scores. Inhibition (*β* = −25.55 [−36.77, −14.26]) also had a significant effect, indicating that lower inhibitory cost was associated with higher output scores. Shifting (*β* = −15.34 [−34.55, 3.04]) did not reach statistical significance in relation to the output scores.

**Table 6:** Results of Bayesian linear regression analysis of Japanese-to-Chinese consecutive interpreting.

Parameter	Comprehension score				Output score			
	EAP	95%[]CI		Rhat	EAP	95%[]CI		Rhat
		Lower	Upper			Lower	Upper	
Intercept	61.77	26.91	98.18	1.00	93.42	79.42	107.59	1.00
Shifting	−17.52	−64.04	30.73	1.00	−15.34	−34.55	3.04	1.00
Updating	11.85	−54.34	76.49	1.00	−29.97	−55.62	−4.53	1.00
Inhibition	−27.17	−55.98	1.29	1.00	−25.55	−36.77	−14.26	1.00

Response times of three executive functions were shifted to “s” for analysis. EAP, expected a posteriori. 95%[]CI, 95% Bayesian credible interval.

#### 4.3.3. Analysis results of Chinese-to-Japanese consecutive interpreting

The results of the Bayesian linear regression analysis indicated that the executive functions had a weak explanatory power for the source language comprehension scores in Chinese-to-Japanese interpreting (*R^2^* = 0.12, 95% CI [0.00, 0.25], adj. *R^2^* = −0.09). However, they exhibited a substantial explanatory power for the target language output scores (*R^2^* = 0.41, 95% CI [0.23, 0.54], adj. *R^2^* = 0.31).

Table 7 provides a summary of the analysis results. In terms of source language comprehension scores, the regression analysis results revealed no significant effect of the three executive functions. Specifically, shifting (*β* = −2.96 [−44.04, 40.20]), updating (*β* = −23.54 [−79.96, 31.89]), and inhibition (*β* = −16.62 [−42.68, 9.09]) did not show a significant impact on the comprehension scores. Conversely, for target language output scores, the analysis indicated that updating (*β* = −45.42 [−71.87, −18.10]) had a significant effect. However, the effects of shifting (*β* = −11.02 [−29.68, 8.14]) and inhibition (*β* = −11.01 [−23.40, 0.90]) were not significant.

**Table 7:** Results of Bayesian linear regression analysis of Chinese-to-Japanese consecutive interpreting.

Parameter	Comprehension score				Output score			
	EAP	95%[]CI		Rhat	EAP	95%[]CI		Rhat
		Lower	Upper			Lower	Upper	
Intercept	95.71	65.26	126.13	1.00	97.74	82.72	112.32	1.00
Shifting	−2.96	−44.04	40.20	1.00	−11.02	−29.68	8.14	1.00
Updating	−23.54	−79.96	31.89	1.00	−45.42	−71.87	−18.10	1.00
Inhibition	−16.62	−42.68	9.09	1.00	−11.01	−23.40	0.90	1.00

Response times of three executive functions were shifted to “s” for analysis. EAP, expected a posteriori. 95%[]CI, 95% Bayesian credible interval.

## 5. Discussion

### 5.1. Effects of executive functions on Japanese-to-Chinese consecutive interpreting

As mentioned earlier, “executive function” refers to the coordination and integration of various cognitive processes to facilitate effective and flexible performance in complex tasks (Diamond, 2013). In this study, we focused on measuring three executive functions that were predicted to be associated with performance in interpreting, based on Miyake et al. (2000)’s “unity and diversity” model. Higher levels of executive function indicate stronger abilities in cognitive control, including attention control and the efficient allocation of cognitive resources. Correlation analysis revealed a weak correlation between the indicators of executive function and the scores of source language comprehension. Similarly, the results of the Bayesian linear regression analysis did not provide sufficient evidence to support a significant impact of the three executive functions on source language comprehension scores in Japanese-to-Chinese consecutive interpreting. These findings can be explained by two possibilities. The first explanation suggests that executive functions may have minimal or no effect on the source language comprehension scores. The second explanation proposes that regardless of the level of executive function, participants prioritize attention to source language comprehension, even those participants with lower levels of executive function.

Upon further consideration, the first explanation appears less likely to be valid in the Japanese-to-Chinese interpreting. Several reasons support this viewpoint. Although from a temporal perspective, the source language comprehension test in this study included integrated scores from both the source language input and target language output stages, it can be argued that understanding the source language is concentrated primarily during the input stage. If there is no understanding of the source language during the input stage, there would be no basis for comprehension during the reformulation and target language output stages. The limited explanatory power of fidelity scores on comprehension scores also indicates a limited contribution of the reformulation stage to overall comprehension. Additionally, the participants in this study were unbalanced bilinguals and the materials were presented in their L2. A study by Wang and Fei (2022) demonstrated that the efficiency of cognitive resource allocation significantly affects source language comprehension in interpreting when the source language is L2. Therefore, it is reasonable to infer that L2 source language comprehension consumes cognitive resources. Individuals with higher levels of executive function may have already completed a substantial amount of comprehension during the source language input stage, while those with lower levels of executive function may continue to rely on the reformulation stage to some extent for source language comprehension, eventually achieving a similar level of understanding as individuals with higher levels of executive function. This could explain why the overall impact of executive function on comprehension scores was not significant.

Considering all of the aforementioned points, it can be inferred that since source language comprehension serves as the foundation for target language output, the absence of significant variations in overall source language comprehension among participants with different levels of executive function implies that participants prioritize the allocation of their limited cognitive resources to L2 source language comprehension (e.g., Lin et al., 2015). This prioritization leads to a decreased allocation of attentional resources toward monitoring the target language output. Consequently, the impact of executive function on target language output scores becomes more significant.

Specifically, the study reveals that the updating function had a significant impact on output scores. Its main role is to monitor information in working memory and promptly replace outdated and no longer relevant information with new information. Higher updating ability was associated with better output performance, which is consistent with previous research findings (e.g., Dong et al., 2018; Liu and Dong, 2020), highlighting the importance of updating in interpreting.

Furthermore, inhibition also demonstrated a significant effect on output scores. Previous research has established the crucial role of inhibition in simultaneous interpreting (e.g., Timarová et al., 2014), and inhibition displayed a strong impact on consecutive interpreting in the present study. In the case of Japanese-to-Chinese consecutive interpreting with unbalanced bilinguals, the strong influence of inhibition on output performance can be attributed to two main factors. Firstly, during the target language output stage, further comprehension of the source language may lead to competition for cognitive resources between the two languages. Secondly, due to the shared Chinese character vocabulary between Chinese and Japanese, non-selective activation of their L1 Chinese is more likely when processing and comprehending L2 Japanese (e.g., Matsumi et al., 2012; Fei et al., 2022). Although consecutive interpreting involves a time lag between source language comprehension and target language output, the output in the target language still relies on the comprehension of L2 Japanese. Hence, inhibition becomes necessary to suppress the non-selective activation of irrelevant information.

In contrast, the study did not find a significant effect of shifting on output scores. Interpreting is a demanding task that involves frequent language switching, and previous research has indicated that interpreter training enhances shifting ability (e.g., Dong and Liu, 2016; Van de Putte et al., 2018; Zhao and Dong, 2020). Therefore, it can be speculated that the effect of shifting may be influenced by the interpreter’s experience of interpreting. Unbalanced bilinguals, who have limitations in both L2 proficiency and interpreting experience, may not demonstrate a significant effect of shifting.

In conclusion, the analysis of target language output in Japanese-to-Chinese consecutive interpreting indicates significant effects in terms of updating and inhibition. This finding suggests that unbalanced bilinguals may not have fully suppressed the competition between the two languages and the non-selective activation of irrelevant information from their L1 during L2 to L1 interpreting. Furthermore, in line with the second explanation for the comprehension test results, it can be inferred that participants tended to prioritize the allocation of cognitive resources to source language comprehension. However, further empirical research is needed to confirm this inference.

### 5.2. Effects of executive functions on Chinese-to-Japanese consecutive interpreting

The findings from the Bayesian linear regression analysis of Chinese-to-Japanese consecutive interpreting did not provide sufficient evidence to establish a significant influence of the three executive functions on the source language comprehension score. When the interpreter’s L1 is used as the source language, their cognitive resource allocation for source language comprehension may be less extensive than the allocation required when the source language is L2 (e.g., Song and Matsumi, 2022). However, as mentioned earlier, this study’s comprehension test included integrated scores from both the source language input and reformulation stages. Therefore, it can not be ruled out entirely that further comprehension of the source language during the reformulation and target language output stages may have led to the non-detection of a significant impact of executive functions.

Exploring this possibility further, a moderate weak positive correlation was found between comprehension scores and fidelity scores of the target language output. Additionally fidelity scores had significantly limited explanatory power for overall comprehension. These findings suggest that even when participants understand the content when listening, they may not interpret it accurately within the given time constraints. The results of Dong and Lin (2013)’s study also indicate a lower likelihood of parallel retrieval of the target language in unbalanced bilinguals when the source language is their L1. Taking into account the results of this study along with previous findings, it can be inferred that the possibility of further comprehension of the source language during the reformulation and target language output stages is low, and the main focus of source language comprehension lies in the input stage. Therefore, in contrast to Japanese-to-Chinese consecutive interpreting, it is likely that the first hypothesis proposed earlier is supported by the findings.

When the source language is L1, the target language output stage may experience cognitive resource deficits (e.g., Dawrant and Setton, 2016; Shang and Li, 2022). Specifically, when generating the target language output based on already-constructed source language representations, efficient and controlled cognitive processing becomes necessary to ensure accurate and fluent output. Thus, executive functions demonstrate a significant effect on output scores. In terms of target language output scores in Chinese-to-Japanese consecutive interpreting, updating demonstrated a significant effect. Chinese-to-Japanese consecutive interpreting imposes a higher cognitive load during the target language output stage due to the utilization of L2 (i.e., Japanese) for output. As a result, faster information updating in working memory becomes crucial for successful output, leading to a pronounced influence of updating.

In contrast to the findings in Japanese-to-Chinese consecutive interpreting, inhibition did not exhibit a significant effect on the target language output in Chinese-to-Japanese consecutive interpreting. With reference to the Revised Hierarchical Model (Kroll and Stewart, 1994), this finding suggests that, when unbalanced bilinguals process their L1, the non-selective activation of L2 is less likely. Moreover, as mentioned earlier, comprehension of the source language in Chinese-to-Japanese interpreting may occur predominantly during the input stage, reducing the possibility of bilingual competition. Although there is a significant moderate negative correlation between inhibition ability and output scores, there is still insufficient evidence to support its significant impact on Chinese-to-Japanese consecutive interpreting output scores. This outcome is consistent with previous studies and supports the explanatory logic of bilingual activation in unbalanced Chinese-Japanese bilinguals (e.g., Matsumi et al., 2012; Fei et al., 2022; Song et al., 2023).

In similarity with the findings on Japanese-to-Chinese consecutive interpreting, there was a significant moderate negative correlation between output scores and switching costs in Chinese-to-Japanese consecutive interpreting. However, the evidence is still insufficient to establish a significant effect of shifting. Previous empirical studies have shown a relationship between shifting ability and interpreting proficiency (e.g., MacNamara et al., 2011). This further supports the speculation that the impact of shifting may be influenced by factors such as the type of interpreting task and the interpreter’s experience of interpreting. Therefore, further investigation of shifting is warranted to provide more empirical evidence, considering factors such as different interpreting tasks (e.g., simultaneous interpreting and consecutive interpreting) and the participants’ experience of interpreting.

By integrating the analysis of source language comprehension and target language output in Chinese-to-Japanese consecutive interpreting, it becomes evident that updating has the most pronounced impact. However, inhibition and shifting do not exhibit significant effects. These findings suggest that the influence of executive functions on the interpreting process is constrained by the direction of the source language.

## 6. Conclusions and limitations

### 6.1. Conclusion

The present study has investigated the influence of three executive functions (shifting, updating, and inhibition) on source language comprehension and target language output in Japanese-to-Chinese and Chinese-to-Japanese consecutive interpreting, involving Chinese-Japanese unbalanced bilinguals. Based on a synthesis of the study’s findings, the following conclusions can be drawn. (1) Updating had a significant impact on both Japanese-to-Chinese and Chinese-to-Japanese consecutive interpreting, indicating that higher updating ability was associated with better interpreting performance. (2) Inhibition exhibited a significant effect on Japanese-to-Chinese interpreting performance, whereas the effect was not significant in Chinese-to-Japanese interpreting. (3) Shifting did not show significant effects in either Japanese-to-Chinese or Chinese-to-Japanese interpreting.

Based on the results of this study, it is recommended that executive function training should be incorporated into interpreter teaching and training programs, with a particular focus on the updating and inhibition functions, in order to enhance the efficiency of cognitive resource allocation for participants.

### 6.2. Limitations and future research

Firstly, as mentioned earlier, this study utilized offline comprehension tests to infer the source language comprehension process. However, it is important to acknowledge that offline tests may not fully capture the real-time dynamics of online comprehension processes. Future research could consider incorporating advanced technologies such as eye-tracking and neurophysiological measures like EEG to provide a more accurate assessment of the source language comprehension process.

Secondly, building upon Miyake et al. (2000)’s “unity and diversity” model, this study examined the effects of three commonly postulated executive functions on the performance of Chinese and Japanese consecutive interpreting. Given that previous research has also suggested that interpreting experience enhances primarily monitoring ability rather than shifting ability (e.g., Babcock and Vallesi, 2017), future studies could further explore the impact of monitoring on interpreting to gain a deeper understanding of its effect. Furthermore, broadening the scope of research to encompass both cool and hot executive functions could yield a more holistic understanding of the executive functions’ implications on interpreting.

Lastly, it would be valuable to explore source languages that impose a higher cognitive load and to incorporate simultaneous interpreting tasks in future studies. Additionally, expanding the research to include professional and student interpreters as participants would allow for a comparative analysis in investigating how executive functions influence the interpreting process. This analysis would provide valuable theoretical insights for interpreter training and contribute to the advancement of interpreter teaching methods.

## Data availability statement

The raw data supporting the conclusions of this article will be made available by the authors, without undue reservation.

## Ethics statement

The studies involving humans were approved by Beijing Center for Japanese Studies, Beijing Foreign Studies University. The studies were conducted in accordance with the local legislation and institutional requirements. The participants provided their written informed consent to participate in this study.

## Author contributions

QS, TS, and XF contributed to the conception of the work. QS and XF collected the experimental data. QS conducted the data analysis and wrote the first manuscript. All authors contributed to the article and approved the submitted version.

## Funding

This study was supported by the “Excellent Talents Program of Beijing Foreign Studies University 2021 (2021-ZYQNJS-011)” and the “2022 Jilin Provincial Social Science Foundation Project, Research on the Writing of ‘Alzheimer’s Disease’ in Contemporary Japanese Literature (2022B111743)”.

## Conflict of interest

The authors declare that the research was conducted in the absence of any commercial or financial relationships that could be construed as a potential conflict of interest.

## Publisher’s note

All claims expressed in this article are solely those of the authors and do not necessarily represent those of their affiliated organizations, or those of the publisher, the editors and the reviewers. Any product that may be evaluated in this article, or claim that may be made by its manufacturer, is not guaranteed or endorsed by the publisher.

## Footnotes

^1^ http://www.chinesereadability.net/CRIE/?LANG=ENG

^2^ https://jreadability.net/en-portal.html

## References

[ref1] Abdullah M. N. S. Karpudewan M. Tanimale B. M. (2021). Executive function of the brain and its influences on understanding of physics concept. Trends. Neurosci. Educ. 24:100159. doi: 10.1016/j.tine.2021.100159 34412861

[ref2] Babcock L. Vallesi A. (2017). Are simultaneous interpreters expert bilinguals, unique bilinguals, or both? Biling. Lang. Congn. 20, 403–417. doi: 10.1017/S1366728915000735

[ref3] Bae M. Jeong C. J. (2021). The role of working memory capacity in interpreting performance: an exploratory study with student interpreters. Transl. Cogn. Behav. 4, 26–46. doi: 10.1075/tcb.00050.bae

[ref4] Bürkner P. C. (2017). Brms: an R package for Bayesian multilevel models using Stan. J. Stat. Softw. 80, 1–28. doi: 10.18637/jss.v080.i01

[ref5] Dawrant A. Setton R. (2016). Conference interpreting: a trainer’s guide. In Conference Interpreting. Amsterdam: John Benjamins Publishing Company.

[ref6] Diamond A. (2013). Executive functions. Annu. Rev. Psychol. 64, 135–168. doi: 10.1146/annurev-psych-113011-143750 23020641 PMC4084861

[ref7] Dong Y. Li P. (2020). Attentional control in interpreting: a model of language control and processing control. Biling. Lang. Congn. 23, 716–728. doi: 10.1017/S1366728919000786

[ref8] Dong Y. Lin J. (2013). Parallel processing of the target language during source language comprehension in interpreting. Biling. Lang. Congn. 16, 682–692. doi: 10.1017/S1366728913000102

[ref9] Dong Y. Liu Y. (2016). Classes in translating and interpreting produce differential gains in shifting and updating. Front. Psychol. 7:1297. doi: 10.3389/fpsyg.2016.01297 27625620 PMC5003826

[ref10] Dong Y. Liu Y. Cai R. (2018). How does consecutive interpreting training influence working memory: a longitudinal study of potential links between the two. Front. Psychol. 9:875. doi: 10.3389/fpsyg.2018.00875 29922199 PMC5996275

[ref11] Dong Y. Wang B. (2013). General v. interpretation-specific language comprehension and production: a two-stage account of the interpreting process. Chinese Transl. J. 1, 19–24.

[ref12] Fei X. Li Y. (2022). An empirical study on the effect of the phonological loop of working memory in simultaneous interpretation. Foreign Language Teaching and Research 3, 455–467. doi: 10.19923/j.cnki.fltr.2022.03.011

[ref13] Fei X. Zhao S. Liu J. (2022). Auditory recognition of Chinese-Japanese cognates and homographs by Chinese JFL learners. Psychologia 64, 1–22. doi: 10.2117/psysoc.2021-A144

[ref14] Gile D. (2009). Basic concepts and models for interpreter and translator training. Amsterdam: John Benjamins Publishing Company.

[ref15] Han X. Fei X. (2021). A study of the cognitive mechanism in consecutive interpreting with reference to working memory capacity and auditory lexical decision speed. Shanghai Journal of Translators 2, 76–81.

[ref16] Harvey P. O. Le Bastard G. Pochon J. B. Levy R. Allilaire J. F. Dubois B. E. E.. (2004). Executive functions and updating of the contents of working memory in unipolar depression. J. Psychiatr. Res. 38, 567–576. doi: 10.1016/j.jpsychires.2004.03.003 15458852

[ref17] Henrard S. Van Daele A. (2017). Different bilingual experiences might modulate executive tasks advantages: comparative analysis between monolinguals, translators, and interpreters. Front. Psychol. 8:1870. doi: 10.3389/fpsyg.2011.00309 29209240 PMC5701671

[ref18] Kline R. B. (2005). Principles and practice of structural equation modeling (2nd ed.). New York: Guilford Press.

[ref19] Kroll J. F. Stewart E. (1994). Category interference in translation and picture naming: evidence for asymmetric connections between bilingual memory representations. J. Mem. Lang. 33, 149–174. doi: 10.1006/jmla.1994.1008

[ref20] Li R. Cheung A. K. Liu K. (2022). A corpus-based investigation of extra-textual, connective, and emphasizing additions in English-Chinese conference interpreting. Front. Psychol. 13:847735. doi: 10.3389/fpsyg.2022.847735 35707653 PMC9190950

[ref21] Li R. Liu K. Cheung A. K. (2023). Interpreter visibility in press conferences: a multimodal conversation analysis of speaker-interpreter interactions. Humanit. Soc. Sciences Commun. 10, 1–12. doi: 10.1057/s41599-023-01974-7

[ref22] Li J. Zhang W. Chen W. (2022). A study on the influence of the visual grammar of consecutive interpreting note-taking on the quality of interpreting. Foreign Lang. Teach. 4, 36–47. doi: 10.13458/j.cnki.flatt.004883

[ref23] Li P. Zhang F. Yu A. Zhao X. (2020). Language history questionnaire (LHQ3): an enhanced tool for assessing multilingual experience. Biling. Lang. Congn. 23, 938–944. doi: 10.1017/S1366728918001153

[ref24] Lin S. (2019). Japanese interpretation exam analysis: Level 3. Beijing: New World Press.

[ref25] Lin J. Dong Y. Cai R. (2015). The hierarchical relation in resource allocation between source language comprehension and language reformulation in interpreting. Foreign Lang. Teach. Res. 47, 447–457.

[ref26] Lin Y. Lv Q. Liang J. (2018). Predicting fluency with language proficiency, working memory, and directionality in simultaneous interpreting. Front. Psychol. 9:1543. doi: 10.3389/fpsyg.2018.01543 30186213 PMC6110880

[ref27] Liu Y. Chen X. Dong Y. (2021). Interpreting and working memory: a developmental view. Foreign Lang. China 5, 55–63. doi: 10.13564/j.cnki.issn.1672-9382.2021.05.007

[ref28] Liu Y. Dong Y. (2020). A longitudinal study of the relationship between early-stage interpreting and working memory. J. Foreign Lang. 43, 112–121.

[ref29] Ma X. (2017). Researches on working memory in interpreting: trends and prospects. Shandong Foreign Lang. Teach. 1, 92–100. doi: 10.16482/j.sdwy37-1026.2017-01-012

[ref30] Macizo P. Bajo M. T. (2004). When translation makes the difference: sentence processing in reading and translation. Psicológica 25, 181–205.

[ref31] Macizo P. Bajo M. T. (2006). Reading for repetition and reading for translation: do they involve the same processes? Cognition 99, 1–34. doi: 10.1016/j.cognition.2004.09.012 16443446

[ref32] MacNamara B. N. Moore A. B. Kegl J. A. Conway A. R. A. (2011). Domain-general cognitive abilities and simultaneous interpreting skill. Interpreting 13, 121–142. doi: 10.1075/intp.13.1.08mac

[ref33] Matsumi N. Fei X. Cai F. (2012). “The lexical processing of Japanese kanji words” in Second language acquisition research and language education. eds. Hatasa K. Hatasa Y. Kudara M. Shimizu T. (Tokyo: Kuroshio Shuppan Press), 43–67

[ref34] McNeish D. (2016). On using Bayesian methods to address small sample problems. Struct. Equ. Model. Multidiscip. J. 23, 750–773. doi: 10.1080/10705511.2016.1186549

[ref35] Miyake A. Friedman N. P. Emerson M. J. Witzki A. H. Howerter A. Wager T. D. (2000). The unity and diversity of executive functions and their contributions to complex “frontal lobe” tasks: a latent variable analysis. Cogn. Psychol. 41, 49–100. doi: 10.1006/cogp.1999.0734 10945922

[ref36] Mizuno A. (2005). Process model for simultaneous interpreting and working memory. Meta 50, 739–752. doi: 10.7202/011015ar

[ref37] Morales J. Padilla F. Gómez-Ariza C. J. Bajo M. T. (2015). Simultaneous interpreting selectively influences working memory and attentional networks. Acta Psychol. 155, 82–91. doi: 10.1016/j.actpsy.2014.12.004 25577491

[ref38] Namba S. Kabir R. S. Miyatani M. Nakao T. (2018). Dynamic displays enhance the ability to discriminate genuine and posed facial expressions of emotion. Front. Psychol. 9:672. doi: 10.3389/fpsyg.2018.00672 29896135 PMC5987704

[ref39] Nour S. Struys E. Woumans E. Hollebeke I. Stengers H. (2020). An interpreter advantage in executive functions? A systematic review. Interpreting 22, 163–186. doi: 10.1075/intp.00045.nou

[ref40] Peirce J. W. (2007). PsychoPy-psychophysics software in Python. J. Neurosci. Methods 162, 8–13. doi: 10.1016/j.jneumeth.2006.11.017 17254636 PMC2018741

[ref41] Pöchhacker F. (2016). Introducing interpreting studies (2nd ed.). London: Routledge.

[ref42] R Core Team (2022). R: a language and environment for statistical computing. R Foundation for Statistical Computing, Vienna, Austria. Available at: http://www.r-project.org/index.html. (Accessed December 31, 2022).

[ref43] Rinne J. O. Tommola J. Laine M. Krause B. J. Schmidt D. Kaasinen V.. (2000). The translating brain: cerebral activation patterns during simultaneous interpreting. Neurosci. Lett. 294, 85–88. doi: 10.1016/S0304-3940(00)01540-8 11058793

[ref44] Rosiers A. Woumans E. Duyck W. Eyckmans J. (2019). Investigating the presumed cognitive advantage of aspiring interpreters. Interpreting 21, 115–134. doi: 10.1075/intp.00022.ros

[ref45] Ruiz J. O. Macizo P. (2019). Lexical and syntactic target language interactions in translation. Acta Psychol. 199:102924. doi: 10.1016/j.actpsy 31465976

[ref46] Ruiz C. Paredes N. Macizo P. Bajo M. T. (2008). Activation of lexical and syntactic target language properties in translation. Acta Psychol. 128, 490–500. doi: 10.1016/j.actpsy.2007.08.004 17884001

[ref47] Shang X. Li D. (2022). A data-driven approach to exploring weighting schemes for assessing bi-directional interpreting performance: evidence from native Chinese-speaking raters. J. Foreign Lang. 45, 82–92.

[ref48] Song Q. (2022). Mechanism of language processing in consecutive interpreting for Chinese-Japanese unbalanced bilinguals: experimental study by manipulating the difficulty of the source language. Invit. Interpreting Transl. Stud. 24, 23–43.

[ref49] Song Q. Fei X. Matsumi N. (2023). The lexical processing of Japanese collocations by Chinese Japanese-as-a-foreign-language learners: an experimental study by manipulating the presentation modality, semantic transparency, and translational congruency. Front. Psychol. 14:1142411. doi: 10.3389/fpsyg.2023.1142411 37082573 PMC10111037

[ref50] Song S. Li D. (2020). The predicting power of cognitive fluency for the development of utterance fluency in simultaneous interpreting. Front. Psychol. 11:1864. doi: 10.3389/fpsyg.2020.01864 32903742 PMC7438941

[ref51] Song Q. Matsumi N. (2022). “Inhibitory function in the processes of consecutive interpreting between Chinese and Japanese” in Studies in education, vol. 3 (Bulletin of the graduate School of Humanities and Social Sciences, Hiroshima University), 48–56.

[ref52] St Clair-Thompson H. L. Gathercole S. E. (2006). Executive functions and achievements in school: shifting, updating, inhibition, and working memory. Q. J. Exp. Psychol. 59, 745–759. doi: 10.1080/17470210500162854 16707360

[ref53] Stroop J. R. (1935). Studies of interference in serial verbal reactions. J. Exp. Psychol. 18, 643–662. doi: 10.1037/h0054651

[ref54] Theodoraki T. E. McGeown S. P. Rhodes S. M. MacPherson S. E. (2020). Developmental changes in executive functions during adolescence: a study of inhibition, shifting, and working memory. Br. J. Dev. Psychol. 38, 74–89. doi: 10.1111/bjdp.12307 31587347

[ref55] Timarová S. Čeňkova I. Meylaerts R. Hertog E. Szmalec A. Duyck W. (2014). Simultaneous interpreting and working memory executive control. Interpreting 16, 139–168. doi: 10.1075/intp.16.2.01tim

[ref56] Tzou Y. Z. Eslami Z. R. Chen H. C. Vaid J. (2012). Effect of language proficiency and degree of formal training in simultaneous interpreting on working memory and interpreting performance: evidence from mandarin-English speakers. Int. J. Biling. 16, 213–227. doi: 10.1177/1367006911403197

[ref57] Van de Putte E. De Baene W. García-Pentón L. Woumans E. Dijkgraaf A. Duyck W. (2018). Anatomical and functional changes in the brain after simultaneous interpreting training: a longitudinal study. Cortex 99, 243–257. doi: 10.1016/j.cortex.2017.11.024 29291529

[ref58] van der Linden L. Van de Putte E. Woumans E. Duyck W. Szmalec A. (2018). Does extreme language control training improve cognitive control? A comparison of professional interpreters, L2 teachers and monolinguals. Front. Psychol. 9:1998. doi: 10.3389/fpsyg.2018.01998 30405488 PMC6206226

[ref59] Wang J. Fei X. (2022). Factors influencing the source language comprehension of unbalanced Chinese-Japanese bilingual students in different directions of consecutive interpretation. J. PLA Univ. Foreign Lang. 45, 121–128.

[ref60] Woumans E. Ceuleers E. Van der Linden L. Szmalec A. Duyck W. (2015). Verbal and nonverbal cognitive control in bilinguals and interpreters. J. Exp. Psychol. Learn. Mem. Cogn. 41, 1579–1986. doi: 10.1037/xlm0000107 25689001

[ref61] Yang C. (2005). Research on interpreting teaching: Theory and practice. Beijing: China Translation Corporation.

[ref62] Yudes C. Macizo P. Bajo T. (2011). The influence of expertise in simultaneous interpreting on non-verbal executive processes. Front. Psychol. 2:309. doi: 10.3389/fpsyg.2011.00309 22059084 PMC3203554

[ref63] Zhang Y. Cheung A. K. (2022). A corpus-based study of modal verbs in Chinese-English governmental press conference interpreting. Front. Psychol. 13:1065077. doi: 10.3389/fpsyg.2022.1065077 36405149 PMC9669789

[ref64] Zhang W. Liu Y. (2023). An empirical investigation of the predictive validity of an interpreting aptitude test battery. Foreign Lang. Teach. 2, 81–92. doi: 10.13458/j.cnki.flatt.004936

[ref65] Zhao H. Dong Y. (2020). The early presence and developmental trend of interpreter advantages in cognitive flexibility: effects from task differences and L2 proficiency. Transl. Cogn. Behav. 3, 241–262. doi: 10.1075/tcb.00043.zha

[ref66] Zhao X. Zhou R. (2011). Sub-function evaluation method of central executive system in working memory. Chin. J. Clin. Psych. 6, 748–752. doi: 10.16128/j.cnki.1005-3611.2011.06.011

[ref68] Zhong J. Zoltan D. Chen Z. (2017). Bayesian inference be introduced to the psychological research: its necessity, ideas and field. J. Psychol. Sci. 6, 1477–1482. doi: 10.16719/j.cnki.1671-6981.20170630

