# Supplementary material for: Effects of executive functions on consecutive interpreting for Chinese-Japanese unbalanced bilinguals
Source: Front Psychol. 2023 Aug 31;14:1236649. doi: 10.3389/fpsyg.2023.1236649 (PMC10506074; doi:10.3389/fpsyg.2023.1236649)
Supplement: Supplementary file 1 [file Data_Sheet_1.pdf]

frontiers

Supplementary Material

Effects of Executive Functions on Consecutive Interpreting for Chinese-Japanese Unbalanced Bilinguals

Qichao Song [1], Ting Song [2], Xiaodong Fei [3*]

[1] Graduate School of Humanities and Social Sciences, Hiroshima University, Japan

[2] School of Foreign Studies, Jilin University, China

[3] Beijing Center for Japanese Studies, Beijing Foreign Studies University, China

*** Correspondence:**
Xiaodong Fei
feixiaodonghi@bfsu.edu.cn

Keywords: Chinese-Japanese unbalanced bilinguals, consecutive interpreting, inhibition, updating, shifting.

1 Japanese-to-Chinese Consecutive Interpreting

segment1: では，これから講座をお聞きいただくにあたって，いかに学習をしていくとよいか，気をつけていただきたいことなどをお話ししましょう。次の三点をお願いしたいと考えております。(Now, let me talk about how you can approach your learning and highlight some important points to consider before attending the lecture. I would like to emphasize the following three points.)

segment2: 1 点目です。講座を聞くという受身の姿勢ではなく，皆さんが自分自身で問いかける姿勢を持ってください。本文の内容，難しい部分，知らなかった語句などに対して自分自身が疑問を持つということです。(Firstly, instead of passively listening to the lecture, I encourage you to adopt an attitude of questioning and inquiry. This means actively engaging with the content, identifying challenging parts, and noting any unfamiliar vocabulary or concepts that arise.)

segment3: できれば講座をお聞きになる前に，あらかじめ一通りは読んでおいて分かりにくいなと思った箇所とか，何か気になった部分などに印をつけておくとよいでしょう。(It would be beneficial to skim through the material beforehand and mark any sections that you find difficult or areas of interest.)

segment4: 2 点目です。メモのできる準備をして講座をお聞きください。お聞きになるなかで「あ～そうか」と気づいたり，「これは大事だな」などと思ったら，必ずメモをしておきましょう。(Secondly, come prepared with the ability to take notes during the lecture. Whenever

you have an "aha" moment or come across something you consider important, be sure to jot it down.)

segment5: 最後に 3 点目。辞書を活用しましょう。国語辞典をできる限りお手元にご用意ください。自分自身で問いかけ，調べてみる，あれこれ考える，はっと気づく，そういう過程で学ぶことがたくさんあるはずです。(Lastly, make use of dictionaries. Please carry a Japanese dictionary with you whenever possible. Through questioning, researching, contemplating, and experiencing those 'aha' moments, you will be able to learn a great deal.)

segment6: それから，学習した事柄を勉強のレベルに止めておくのではなく，実生活で試したり，活用したりしてほしいということです。例えば，自己紹介の仕方を学んだら，クラブに入部する時や何かの集まりに初めて参加する時などの場面でぜひ試してみてほしいと思います。(Furthermore, I encourage you to go beyond studying and actually apply what you have learned in real-life situations. For example, if you learn a new way of introducing yourself, try it out when joining a club or participating in a gathering for the first time.)

2 Chinese-to-Japanese Consecutive Interpreting

segment1: 现在，年轻人都玩手机，还用它买衣服，吃的，用的啊等等，干什么都喜欢使用手机。只要自己选好想要的东西，轻轻一点，交易就算完成了，你说这手机方便不方便？(Nowadays, young people are all using their mobile phones for everything, from buying clothes to ordering food and making various other transactions. They prefer using their mobile phones for everything. With just a gentle touch after selecting the desired item, the transaction is completed. Can you imagine how convenient these phones are?)

segment2: 而今年还能够使用手机购买车票了，这样一来，使用互联网，用手机来购买车票的人肯定会不少。看来，这种购买车票的方式真是受到大家的喜爱。(And this year, you can even purchase train tickets with your mobile phone. With this option, using the Internet and mobile phones to buy train tickets will certainly become popular. It seems that this way of purchasing tickets is really loved by everyone.)

segment3: 目前，自助售票机已经被移出了售票大厅，进入了校园，超市，社区等。而且也推出了售票窗口和自助售票机的扫码支付服务。(Currently, self-service ticket machines have been removed from ticket halls and are now available on campuses, supermarkets, communities, and other places. In addition, ticket windows and self-service ticket machines have introduced QR code payment services.)

segment4: 这样的方式，既提高了售票的速度，又简化了购买车票的流程，实在是太方便了。国家真的是处处都为大家着想，生活在这个年代里真的让我们觉得十分幸福。(This approach not only speeds up the ticketing process but also simplifies the ticket-purchasing procedure. It is truly

convenient. The country is really considerate in every aspect, and living in this era makes us feel very fortunate.)

segment5: 就连买车票这样的小事，国家都为大家做好了打算，提供如此方便的服务，那么别的服务能不好吗？我们从心里感到高兴。(Even for small matters like buying train tickets, the country has made plans and provided such convenient services for everyone. If even services like purchasing train tickets can be this good, can other services be bad? We feel delighted from the bottom of our hearts.)

***Notes*:** It is worth highlighting that Japanese text tends to be around 1.5 times longer than its Chinese counterpart when conveying equivalent content. This aspect was carefully considered during the selection of the source language for the present study. The sentence count (18) in the Chinese text analysis results derived from CRIE 3.0 does not align with the sentence count (10) calculated based on punctuation marks (i.e., periods and question marks). We reported the findings derived from CRIE 3.0 analyses.
